# Posterior cerebral atrophy in the absence of medial temporal lobe atrophy in pathologically-confirmed Alzheimer's disease

**DOI:** 10.1016/j.neurobiolaging.2011.04.003

**Published:** 2012-03

**Authors:** Manja Lehmann, Esther L.G.E. Koedam, Josephine Barnes, Jonathan W. Bartlett, Natalie S. Ryan, Yolande A.L. Pijnenburg, Frederik Barkhof, Mike P. Wattjes, Philip Scheltens, Nick C. Fox

**Affiliations:** a Dementia Research Centre, UCL Institute of Neurology, Queen Square, London, UK; b Alzheimer Centre and Department of Neurology, VU University Medical Centre, Amsterdam, the Netherlands; c Department of Radiology, VU University Medical Centre, Amsterdam, the Netherlands

**Keywords:** Visual rating scales, Posterior atrophy, Medial temporal lobe atrophy, MRI, Dementia, Pathology, Manual volumes

## Abstract

Medial temporal lobe atrophy (MTA) is a recognized marker of Alzheimer's disease (AD), however, it can be prominent in frontotemporal lobar degeneration (FTLD). There is an increasing awareness that posterior atrophy (PA) is important in AD and may aid the differentiation of AD from FTLD. Visual rating scales are a convenient way of assessing atrophy in a clinical setting. In this study, 2 visual rating scales measuring MTA and PA were used to compare atrophy patterns in 62 pathologically-confirmed AD and 40 FTLD patients. Anatomical correspondence of MTA and PA was assessed using manually-delineated regions of the hippocampus and posterior cingulate gyrus, respectively. Both MTA and PA scales showed good inter- and intrarater reliabilities (kappa > 0.8). MTA scores showed a good correspondence with manual hippocampal volumes. Thirty percent of the AD patients showed PA in the absence of MTA. Adding the PA to the MTA scale improved discrimination of AD from FTLD, and early-onset AD from normal aging. These results underline the importance of considering PA in AD diagnosis, particularly in younger patients where medial temporal atrophy may be less conspicuous.

## 1 Introduction

Alzheimer's disease (AD) is the most common cause of dementia (Hebert et al., 2003). Frontotemporal lobar degeneration (FTLD), although less prevalent overall, is almost as common as AD in patients under the age of 65 years (Harvey et al., 2003; Ratnavalli et al., 2002). AD and FTLD are histopathologically distinct, with AD being characterized by extracellular amyloid plaques and intraneuronal neurofibrillary tangles (Braak and Braak, 1991), and FTLD by the presence of non-AD histological pathology, most commonly either tau-positive inclusions or ubiquitin-positive, TAR DNA-binding protein (TDP)-43-positive inclusions (Cairns et al., 2007).

Clinically, AD and FTLD may present with overlapping symptoms, in particular in the early stages of the disease. AD typically presents with episodic memory impairment which progresses to involve multiple cognitive domains (McKhann et al., 1984). Less common “atypical” forms of AD have been described in which memory is not the primary deficit. These patients may present with visuospatial and visuoperceptual problems (Benson et al., 1988), while others present with behavioral symptoms (Johnson et al., 1999; Taylor et al., 2008), yet others have predominantly language difficulties (Gorno-Tempini et al., 2008). Patients with FTLD pathology may also present with a range of different clinical symptoms, most commonly categorized into behavioral-variant frontotemporal dementia (bvFTD), and the progressive aphasias of semantic dementia (SD), and progressive nonfluent aphasia (PNFA) (Neary et al., 1998).

Structural brain changes (atrophy) mirror the pathological patterns in these disease groups and can be visualized in life using magnetic resonance imaging (MRI) (Likeman et al., 2005). In AD, medial temporal lobe atrophy (MTA) on MRI is frequently an early feature of the disease, with greatest volume loss found in the hippocampus, entorhinal cortex, amygdala, and parahippocampus (Basso et al., 2006; Fox and Schott, 2004; Teipel et al., 2006). In FTLD, regions most affected are the frontal and anterior temporal lobes in bvFTD, left anterior temporal lobe in SD, and left perisylvian fissure in PNFA (for a review see Seelaar et al., 2011). Therefore, while the presence of MTA is an important hallmark of AD and the new AD research criteria (Dubois et al., 2007) propose that the presence of memory loss with MTA are sufficient to make a diagnosis of prodromal AD, MTA is also present in FTLD and normal aging (Chan et al., 2001; Fjell et al., 2009; Frisoni et al., 1996; Galton et al., 2001a, 2001b). An increasing number of studies emphasize the presence of atrophy in posterior areas of the brain such as precuneus and posterior cingulate gyrus in AD (Jones et al., 2006; Karas et al., 2007). Atrophy in these regions has further been suggested to be more of a feature of early-onset AD (EOAD) than late-onset AD (LOAD) (Frisoni et al., 2007; Ishii et al., 2005a; Shiino et al., 2008).

Visual rating scales are increasingly used to assess atrophy for routine clinical use (Scheltens et al., 1992, 1997). The MTA scale has been shown to discriminate well between AD and healthy controls, and to predict the conversion from mild cognitive impairment (MCI) to AD (Korf et al., 2004; Scheltens et al., 1992, 1995). We have recently developed a visual rating scale for posterior atrophy (PA), which includes the posterior cingulate gyrus, precuneus, and parietal lobes.

Because the new diagnostic criteria may be used for clinical trials which specifically target AD pathology, it is only with pathologically-proven cases that one can be sure of the predictive value of different atrophy measures such as MTA or PA ratings. The current study therefore aimed (1) to assess the inter- and intrarater agreement of the MTA and PA scale in a large cohort of pathologically-proven AD and FTLD patients, and controls; (2) to investigate associations between visual rating scores and manual volumetric measures; (3) to assess the utility of MTA and PA scales in distinguishing between pathologically-confirmed AD and controls as well as FTLD which may be misdiagnosed as AD; and (4) to assess the discriminatory ability of visual ratings according to age at onset.

## 2 Methods

### 2.1 Subjects

Patients were selected retrospectively from a database of pathologically- and genetically-confirmed subjects. This identified 152 subjects who had undergone volumetric magnetic resonance (MR) imaging: 62 patients with “definite” AD (55 pathologically- and 7 genetically-confirmed) according to National Institute of Neurological and Communicative Disorders and Stroke-Alzheimer's Disease and Related Disorders Association (NINCDS-ADRDA) criteria (McKhann et al., 1984), and 40 patients with FTLD (all pathologically-confirmed). Fifty healthy age- and gender-matched controls were also included. Subject demographics are summarized in Table 1. All patients had attended the Cognitive Disorders Clinic at the National Hospital for Neurology and Neurosurgery, London, UK. Informed consent was obtained from all subjects and the study had local ethics committee approval. We excluded subjects with mixed AD and dementia with Lewy bodies (DLB) pathology. Patients underwent comprehensive clinical assessment which included the Mini-Mental State Examination (MMSE) (Folstein et al., 1975). The clinical notes were retrospectively reviewed by a neurologist to determine whether the clinical presentation was predominantly an amnestic, behavioral, language presentation, or a posterior cortical atrophy (PCA) syndrome, characterized by deficits in higher visual processing skills, calculation, and/or praxis.

### 2.2 MRI acquisition and processing

T1-weighted volumetric MR scans were performed on 1.5 Tesla Signa units (General Electric, Milwaukee, WI, USA) using a volumetric spoiled gradient recalled (SPGR) sequence with 1.5-mm thick slices covering the head. All scans were spatially normalized into Montreal Neurological Institute (MNI) 305 atlas space (Mazziotta et al., 1995) with a 6 degrees-of-freedom (dof) registration.

### 2.3 Regional volumes in subset of subjects

Manual delineations of the hippocampus and posterior cingulate gyrus had been obtained in a subset of 38 subjects (11 controls, 19 AD, 8 FTLD, matched for age and gender) as part of a previous study (Barnes et al., 2007). Demographic details as well as manual delineation protocols are described in Barnes et al. (2007). Volumes in mm^3^ were measured and analyzed separately for the left and right hemisphere. Total intracranial volumes (TIVs) were also available for the same subjects derived according to a previously described protocol (Whitwell et al., 2001).

### 2.4 Visual rating scales

All scans were assessed by 2 raters (EK and ML) blinded to diagnoses and clinical information. MTA and PA were rated once by each rater for the whole cohort (152 subjects), and twice for the subset of 38 subjects for which there were cingulate and hippocampal volumes available.

#### 2.4.1 MTA scale

MTA was assessed using a standardized scale (Scheltens et al., 1992). T1-weighted images were viewed in the coronal plane and scores for the left and right hemispheres were recorded. The scale rates atrophy on a 5-point scale (0 = absent, 1 = minimal, 2 = mild, 3 = moderate, and 4 = severe) based on the height of the hippocampal formation and the width of the choroid fissure and the temporal horn.

#### 2.4.2 PA scale

PA was scored on T1-weighted images viewed in the sagittal, axial, and coronal planes. Separate scores for the left and right hemispheres were obtained. The following anatomical features were rated in 3 different orientations:

(1) Sagittal plane: Evaluation of widening of the posterior cingulate and parieto-occipital sulcus, and atrophy of the precuneus in the paramedian-sagittal plane;
(2) Axial plane: Evaluation of widening of the posterior cingulate sulcus and sulcal dilatation in the parietal lobes; and
(3) Coronal plane: Evaluation of widening of the posterior cingulate sulcus and parietal sulci.

**Fig. 1:**
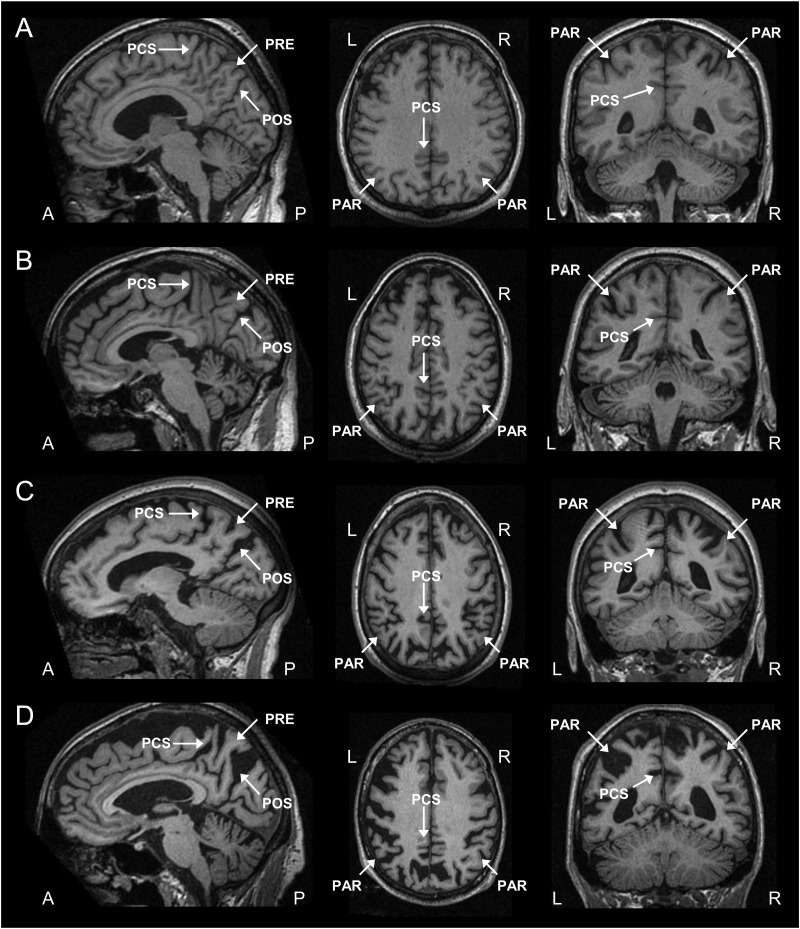
T1-weighted sagittal, axial, and coronal images as examples for each grade of the posterior atrophy (PA) scale. (A) Grade 0 = no atrophy; (B) Grade 1 = minimal atrophy; (C) Grade 2 = moderate atrophy; and (D) Grade 3 = severe atrophy. PAR, parietal lobe; PCS, posterior cingulate sulcus; POS, parieto-occipital sulcus; PRE, precuneus.

The PA scale rates atrophy on a 4-point scale: Grade 0 represents closed posterior cingulate and parieto-occipital sulci and closed sulci of the parietal lobes and precuneus (Fig. 1A); Grade 1 includes a mild widening of the posterior cingulate and parieto-occipital sulci, with mild atrophy of the parietal lobes and precuneus (Fig. 1B); Grade 2 shows substantial widening of the posterior cingulate and parieto-occipital sulci, with substantial atrophy of the parietal lobes and precuneus (Fig. 1C); and Grade 3 represents end-stage atrophy with evident widening of the posterior cingulate and parieto-occipital sulci and knife-blade atrophy of the parietal lobes and precuneus (Fig. 1D). When there was a difference between scores in the different planes (e.g., score 1 for the sagittal view and score 2 for the axial view), the highest score was given.

### 2.5 Statistical analysis

**Fig. 2:**
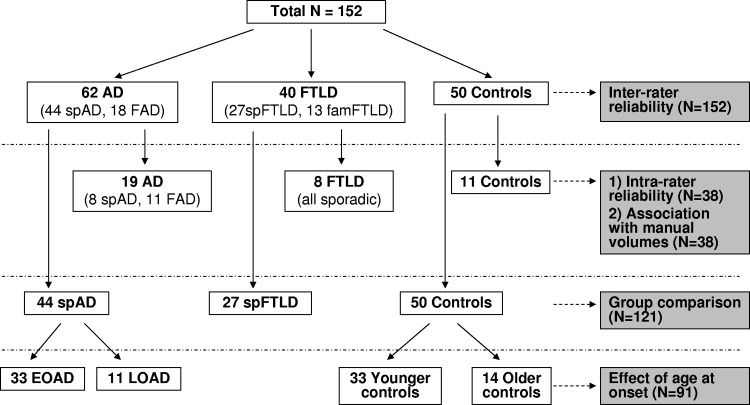
Overview of subject groups included for each analysis. EOAD, early-onset Alzheimer's disease; FAD, familial Alzheimer's disease; famFTLD, familial frontotemporal lobar degeneration; LOAD, late-onset Alzheimer's disease; spAD, sporadic Alzheimer's disease; spFTLD, sporadic frontotemporal lobar degeneration.

All statistical analyses were performed using Stata version 11 (Stata Corp., College Station, TX, USA). A flow chart showing which subjects were used for the different parts of the analysis is presented in Fig. 2.

#### 2.5.1 Inter- and intrarater reliability

Interrater reliability between the 2 raters for the MTA and PA scales was assessed using scores from the whole cohort of 152 subjects whereas intrarater reliability for both scales was assessed in the subset of 38 subjects. Reliability was measured using quadratically weighted kappas (Cohen, 1968). Bias-corrected and accelerated 95% bootstrap confidence intervals (CI) for kappa were found using 10,000 bootstrap samples.

#### 2.5.2 Anatomical correlates

Relationships between volumes and rating scores were assessed by calculating mean volumes (in mm^3^) and SDs of hippocampal volumes for each MTA grade, and posterior cingulate gyrus volumes for each PA grade. Ratings of both raters were averaged, resulting in half grades (i.e., 0.5, 1.5, etc.). In order to ensure reasonable subject numbers for each grade, volumes were averaged for Grades 0 and 0.5, 1 and 1.5, 2 and 2.5, and all ≥ 3. Furthermore, MTA and PA scores were dichotomized into normal and abnormal scores, with a score of > 1 being considered abnormal. Mean volumes for normal and abnormal scores as well as percent differences between normal and abnormal are presented. To assess whether differences between grades were statistically significant, an ordinal logistic regression was performed with the rating score (MTA and PA) as dependent variable and corresponding volume (hippocampal and posterior cingulate gyrus) as independent variable, adjusting for age, gender, and TIV.

#### 2.5.3 Group comparisons

MTA and PA rating scores were first analyzed in pathologically-confirmed sporadic AD (*n* = 44) and compared with pathologically-confirmed sporadic FTLD (*n* = 27) and controls (see Fig. 2). In order to assess the effect of age at onset on visual ratings, the sporadic AD group was split into pathologically-confirmed EOAD (age of onset < 65 years, *n* = 33) and pathologically-confirmed LOAD (age of onset ≥ 65 years, *n* = 11), and compared with younger (*n* = 33) and older controls (*n* = 14), matched for age at time of scan and gender to the EOAD and LOAD groups, respectively. Differences between early- and late-onset FTLD patients were not examined because the number of late-onset FTLD was too small (*n* = 4) to conduct a meaningful comparison.

##### 2.5.3.1 Atrophy patterns

Mean scores and SDs of MTA and PA ratings were calculated and differences in scores between groups were assessed using a Mann-Whitney/Wilcoxon rank-sum test. MTA and PA scores were further dichotomized into normal and abnormal, with a score of >1 being considered abnormal. Ratings of both raters were averaged.

##### 2.5.3.2 Classification analysis and added value

In order to assess the diagnostic accuracy of the MTA and PA scales for discriminating between groups, we estimated the area under the receiver operator curve (ROC), denoted AUC. The AUC measures the ability of a score to discriminate between groups, and ranges from 0.5 (no predictive value) to 1 (perfect discrimination). The added value of MTA and PA was assessed by combining MTA and PA in a logistic regression model. Differences in AUCs between combined and single rating scales are reported as well as 95% Wald-type CIs (1000 bootstrap samples) and *p*-values based on a *z*-test of the difference in AUCs, using the bootstrap standard error. This was performed with the user-written Stata command comproc (Janes et al., 2009). These analyses were also based on the average of ratings from 2 raters.

## 3 Results

### 3.1 Subjects

Subject demographics are presented in Table 1. Of the 62 AD patients, 48 had a postmortem confirmation of AD, 7 had had a brain biopsy, and 7 had diagnostic genetic testing alone. The AD group consisted of 44 sporadic cases, and 18 familial cases. All sporadic AD patients had pathological confirmation of disease. Of the 44 sporadic AD cases, 19 patients had a typical amnestic presentation during life, whereas 10 had a PCA syndrome, 9 had a language presentation, and 4 had a behavioral presentation. There were insufficient clinical details to determine the presenting clinical features for 2 subjects. Furthermore, of the 44 sporadic cases, 33 patients had an age at onset < 65 years (EOAD), whereas 11 had an age at onset of ≥ 65 years (LOAD). All of the familial AD subjects had an amnestic presentation during life. Of the 18 familial AD subjects, 11 had both genetic testing and postmortem confirmation of disease, and 7 had a genetic diagnosis only. The familial AD cohort comprised 9 individuals with a presenilin 1 mutation and 8 with an amyloid precursor protein (APP) gene. One individual had pathologically-confirmed AD but screened negative for mutations currently known to cause familial AD. As she had a strong family history suggesting autosomal dominant inheritance of AD and a very young age at symptom onset of 36, this subject was included in the familial cohort.

Of the 40 FTLD patients included in this study, 36 had postmortem confirmation of FTLD, and 4 had a brain biopsy. Twenty of the FTLD cases had a behavioral syndrome during life, and 20 had a language-led clinical presentation. Twenty-seven patients were sporadic cases, whereas 13 had a family history of FTLD. Of the 27 sporadic cases, 9 were tau-positive (Pick's pathology) and 18 were tau-negative (13 TDP1, 3 ubiquitin-positive with unknown TDP status, 1 with neuronal intermediate filament inclusion disease [NIFID], and 1 with dementia lacking distinctive histology [DLDH]). Twenty-three had an age of onset < 65 years, and 4 had an age of onset of ≥ 65 years. Of the 13 familial cases, 7 were tau-positive and 6 were ubiquitin-positive (5 TDP43-positive and 1 TDP43-negative). Seven familial patients had a microtubule-associated protein tau (MAPT) mutation, 3 had a progranulin (PGRN) mutation, and in 3 subjects with a history suggestive of FTLD a genetic mutation was not identified.

There was no evidence of differences across the 3 main groups (controls, AD, FTLD) for age and gender, and for the FTLD and AD groups no significant difference in distribution of sporadic and familial cases, age of disease onset, disease duration, or time to death (Table 1). However, the AD subjects had a lower MMSE than the FTLD group (*p* < 0.0001). Scanner distribution (i.e., number of scans from different scanners used in each group) was not significantly different between AD and FTLD (*p* = 0.3), however, the controls had a greater number of scans on scanner A compared with AD and FTLD (*p* = 0.02).

There was no significant difference in age or gender between sporadic EOAD (mean age [SD]: 58.8 [6.6] years, 61% male) and younger controls (mean age [SD]: 56.5 [7.3] years, 58% male). Similarly, there was no evidence of a difference in age or gender between LOAD (mean age [SD]: 73.0 [5.4] years, 64% male) and older controls (mean age [SD]: 72.7 [5.0] years, 64% male). The EOAD and LOAD groups had a mean MMSE score of 16.7 and 19.1, respectively (*p* = 0.5), and a mean disease duration of 4.2 years and 2.8 years, respectively (*p* = 0.02).

### 3.2 Inter- and intrarater reliability

Both the MTA and PA scales had good inter- and intrarater reliability. Interrater kappa scores for the MTA scale were 0.88 for left, 0.86 for right, and 0.91 for mean of both hemisphere scores (Table 2). Interrater kappa scores for the PA scale were 0.83 for left, 0.82 for right, and 0.84 for mean of left and right hemisphere scores. Intrarater kappa scores ranged from 0.83 to 0.91 for the MTA, and from 0.87 to 0.89 for the PA scale.

### 3.3 Anatomical correlates

Higher MTA scores were associated with smaller hippocampal volumes in both hemispheres (*p* = 0.001, Table 3). Lower posterior cingulate gyrus volumes were associated with higher PA ratings, which was statistically significant in the right hemisphere (*p* = 0.004), but not in the left hemisphere (*p* = 0.3). Comparing the proportion of explained variation (*R*^2^) in posterior cingulate volumes for PA ratings versus dichotomized (present/absent) PA scores revealed that the actual PA ratings explain relatively little variability in volumes over and above that explained by the present/absent PA ratings.

Mean hippocampal volume of subjects with abnormal MTA scores (> 1.5) was 21.0% lower than for those with normal MTA scores in the left hemisphere (1992 mm^3^ versus 2520 mm^3^), and 17.5% lower in the right hemisphere (2214 mm^3^ versus 2685 mm^3^). Similarly, dichotomizing PA scores into normal and abnormal, mean posterior cingulate gyrus volume of subjects with abnormal PA scores was 21.7% lower than for those with normal PA ratings in the left (1936 mm^3^ for abnormal and 2471 mm^3^ for normal), and 23.3% lower in the right hemisphere (2149 mm^3^ for abnormal and 2800 mm^3^ for normal).

### 3.4 Visual ratings in AD and FTLD

#### 3.4.1 Atrophy patterns

MTA ratings were significantly greater in the FTLD group compared with controls (*p* < 0.0001) and compared with AD (*p* = 0.002 for left hemisphere; *p* = 0.03 for mean left and right, Table 4). MTA ratings were also significantly higher in the AD group compared with controls (*p* < 0.0001). Ratings for the PA scale were greater in the AD group compared with controls (*p* < 0.001) and FTLD (*p* = 0.004 for right hemisphere; *p* = 0.02 for mean left and right). PA ratings were also significantly higher in the FTLD group compared with controls in the left hemisphere (*p* = 0.03).

Dichotomizing MTA and PA scores into normal and abnormal revealed different patterns of atrophy in each group (Table 5). The majority (72%) of the control subjects had normal atrophy scores (i.e., normal MTA and PA); 30% of the AD patients had PA in the absence of abnormal MTA (i.e., abnormal PA, normal MTA), whereas only 7% of the FTLD group had abnormal PA score and normal MTA. Sixty-three percent of the FTLD patients had a normal PA with an abnormal MTA score. The demographic data for the 4 different AD subgroups (i.e., no atrophy, MTA only, PA only, both MTA and PA; see Supplementary Table 1) further show that the PA only AD group is slightly younger in age than AD subjects with MTA only, however, this difference was not statistically significant.

#### 3.4.2 Classification analysis

While discrimination abilities for separating AD from control subjects were good for both MTA and PA scales (AUC 0.80 and 0.74, respectively, for mean left and right), they improved significantly when combining both scales (0.87 mean left and right, Table 6). In contrast, adding the PA to the MTA scale did not improve accuracy for separating FTLD from controls (*p* = 0.4). For the discrimination between AD and FTLD, combining both scales improved classification accuracy to 0.73 (mean left and right), although the differences were not statistically significant. The greatest improvement was found in the right hemisphere, where adding the PA to the MTA scale significantly increased accuracy to 0.72 (*p* = 0.02).

### 3.5 The effect of onset on visual ratings in AD

#### 3.5.1 Atrophy patterns in EOAD and LOAD

Both EOAD and LOAD showed significantly higher MTA scores compared with younger and older controls, respectively, with MTA scores being significantly higher in LOAD than EOAD (*p* = 0.02 for right, *p* = 0.04 for mean left and right, Table 7). In addition, EOAD showed significantly greater PA scores compared with younger controls (*p* = 0.0001), whereas no significant difference in PA scores between LOAD and older controls was found.

Dichotomizing MTA and PA scores into normal and abnormal revealed that over 1 third of EOAD patients (33%) had PA only (Table 8). Of the LOAD patients, all had some abnormal atrophy score with almost half of the LOAD patients having MTA only (46%). The majority of the younger controls (81%) had no atrophy, whereas almost half of the older controls (43%) had either MTA, PA, or both MTA and PA. These data suggest that abnormal PA scores may be more useful in younger patients and may be less specific in elderly people.

#### 3.5.2 Classification analysis

Combining MTA and PA ratings significantly improved the separation of EOAD from younger controls to 0.89 (mean left and right, Table 9). In contrast, adding the PA to the MTA scale did not significantly improve separation of LOAD from older controls (*p* = 0.5 for mean left and right), whereas adding the MTA to the PA scale did result in a better separation (*p* = 0.01 for mean left and right). Discriminatory ability of the MTA scale was higher for the separation of LOAD from older controls than EOAD from younger controls, whereas the PA scale showed a better discrimination for the EOAD from younger control classification than LOAD versus older controls. While these differences were not statistically significant, the magnitude of these differences, in particularly for the PA scale, was relatively high.

## 4 Discussion

This study demonstrates that in addition to medial temporal lobe atrophy being a feature of AD, prominent atrophy in posterior regions of the brain is also frequently found. Furthermore, in a subset of patients with pathologically-proven AD, posterior atrophy may be present in the absence of marked atrophy in the medial temporal lobes. Because atrophy in the medial temporal lobes is a suggested marker for AD in new diagnostic criteria, a substantial proportion of AD patients may be misdiagnosed as normal if only this area is considered. Furthermore, MTA is also common in FTLD which may lead to misdiagnosis of FTLD patients as AD. However, PA is less common in FTLD, making it a potentially useful marker to separate AD and FTLD patients. In our study we show that the presence of posterior atrophy helps to distinguish AD from FTLD, and also helps separate early-onset AD patients (onset < 65 years) from younger controls. This study, with the strength of pathological confirmation in all patients, therefore suggests that the presence of posterior atrophy may be a useful additional marker of AD pathology, over and above MTA. Our investigation further presents and establishes a tool with which posterior atrophy can be easily and reliably assessed in a clinical setting.

Nonmemory related deficits such as visuospatial problems are increasingly recognized as being a feature of AD (van der Flier et al., 2011). It is perhaps therefore not surprising that an anatomical correlate of visuospatial deficits, namely posterior atrophy, is useful in distinguishing AD from FTLD. The involvement of posterior brain regions in AD seen in this study is in accordance with previous reports showing that AD is not only characterized by atrophy of the medial temporal lobe but also of posterior regions such as precuneus and posterior cingulate gyrus (Barnes et al., 2007; Frisoni et al., 2007; Galton et al., 2000). Posterior hypometabolism has long been recognized to be characteristic of AD (Ishii et al., 2005b; Minoshima et al., 1997). Posterior regions have also been shown to have higher levels of amyloid deposition early in AD (Kemppainen et al., 2007; Klunk et al., 2004). Functional imaging studies have further shown that the default-mode network, which includes medial temporal lobe regions and posterior regions, is also affected early in AD (Greicius et al., 2004; Zhang et al., 2010).

Both the MTA and PA scales showed reasonable inter- and intrarater kappa scores. Higher MTA scores further corresponded with smaller hippocampal volumes, similar to previous studies (Bresciani et al., 2005; Scheltens et al., 1992; Wahlund et al., 2000). The difference in hippocampal volumes between normal and abnormal MTA score was relatively high, with the mean of abnormal volumes being 21.0% lower in the left and 17.5% lower in the right hemisphere than the mean of normal volumes, with on average a 1 point rise in MTA score being equivalent to around 12% reduction in hippocampal volume. It should be noted, however, that this difference is determined by the cutoff score used to define normal and abnormal MTA. It has been shown that an appropriate cutoff score is age-dependent, with a score of > 1 being considered abnormal below the age of 75, whereas in patients > 75 years of age a score of > 2 would be required to identify abnormal MTA (Scheltens et al., 1992). Duara et al. showed that a mean MTA score of 1.33 produced optimal sensitivities and specificities to separate patients with AD and mild cognitive impairment (MCI) from healthy controls (Duara et al., 2008). Using postmortem MRI of very old AD patients (> 85 years), Barkhof et al. showed that a cutoff score of 2 correctly excluded subjects with no or borderline Alzheimer-type pathology (Barkhof et al., 2007). Because the AD group in the current study is relatively young (mean age 58 years), a cutoff score of > 1 was used to define abnormal MTA. To ensure consistency the same cutoff score was used for the PA scale. Higher PA scores only roughly corresponded with smaller volumes of the posterior cingulate gyrus, probably because the PA scale reflects more than just posterior cingulate atrophy. Although there was only evidence of an association in the right hemisphere, a test for interaction showed no evidence that the association differs by hemisphere (*p* = 0.1).

The presence of MTA in the FTLD group underlines the fact that hippocampal atrophy is not exclusive to AD, but has been widely described in FTLD as well (Chan et al., 2001; Galton et al., 2001a, 2001b; Mesulam, 2001; Thompson et al., 2003). Posterior atrophy scores, on the other hand, were significantly higher in the AD group compared with FTLD. This difference was driven by greater posterior atrophy in the right hemisphere in AD. It is perhaps surprising that a proportion of FTLD cases have quite prominent posterior atrophy in the left hemisphere. This is likely to be due to the language cases in this group where left-sided atrophy can spread posteriorly as the disease progresses. Higher PA ratings in the right hemisphere in AD significantly improved the distinction of AD from FTLD. It should be noted, however, that average scores of 2 raters were used in this study. Classification accuracies therefore may not necessarily reflect performance of a single rater.

Interestingly, 30% of AD subjects in this study had marked PA in the absence of an abnormal MTA rating, whereas only 18% showed the opposite pattern. The AD patients with PA only tended to be younger (in terms of age and onset) than patients with MTA only. Splitting the AD group into early- and late-onset patients further revealed that PA ratings significantly improved the classification of EOAD from younger control subjects, whereas it did not improve the separation between LOAD and older control subjects. This suggests that atrophy in posterior regions of the brain is particularly important to consider when making a diagnosis in early-onset patients. However, this segregation with age is not exclusive; some younger AD patients may present with mostly MTA, while some LOAD may have mostly PA.

One potential limitation is the variety in image acquisition. It is unclear how MTA and PA ratings are affected by different scan acquisition protocols and quality. However, it has been shown that MTA ratings are comparable using MRI and computed tomography (CT) (Wattjes et al., 2009). Furthermore, the fact that strong patterns were still detected in different groups suggests that visual rating tools are relatively robust to varying image quality. It should further be noted that only sporadic AD and FTLD cases were included in the group comparison and classification analyses. This was motivated by the fact that the familial AD cases were significantly younger than the sporadic AD cases (mean age 47.7 [SD 6.4] for familial AD and 62.3 [SD 8.9] for sporadic AD), and that previous studies suggesting that familial AD patients can have a different clinical phenotype and different patterns of amyloid accumulation than sporadic AD cases (Knight et al., 2011; Ryan and Rossor, 2010). The exclusion of the familial cases therefore resulted in more homogenous groups for the group comparison analysis. While some of these AD patients had nonamnestic clinical presentations during life and may therefore be considered as “atypical”, all of these patients had pathological confirmation of disease. Because pathological confirmation remains the gold standard to establish a definite diagnosis of AD, and because potential interventional treatments are likely to specifically target AD pathology, the main objective of the current study was to assess visual ratings in patients with AD pathology irrespective of clinical diagnosis. Further investigations assessing the relative utility of the visual assessment tools presented in this study should be performed in clinically-diagnosed older sporadic AD patients.

In summary, the current study demonstrates pronounced atrophy in posterior cerebral regions in the absence of clear atrophy in the medial temporal lobe in patients with pathologically-confirmed AD. Because MTA is currently a proposed diagnostic marker for AD, these findings may suggest that some AD patients may not receive a diagnosis of AD if only MTA is considered. The presence of posterior atrophy may be a helpful additional marker for AD, especially in younger patients. This study further presents a tool with which posterior cerebral atrophy can be easily and reliably assessed in a clinical setting. Visual ratings of PA may improve diagnostic accuracy for distinguishing AD from FTLD, and may be valuable in distinguishing early-onset AD from younger control subjects.

## Disclosure statement

None of the authors have any disclosures to make.

Informed consent was obtained from all subjects and the study had local ethics committee approval.

## Figures and Tables

**Table 1:** Subject demographics

	Control	AD	FTLD	*p*
*n*	50	62	40	NA
Age in years, mean (SD)	59.7 (11.3)	58.2 (10.6)	59.2 (8.9)	0.7^a^
Gender, male/female	29/21	34/28	26/14	0.6^b^
Sporadic/familial	NA	44/18	27/13	0.8^b^
MMSE, mean (SD)^c^	29.1 (1.1)	17.2 (6.8)	22.9 (5.2)	<0.0001^a^
Age at onset in years, mean (SD)	NA	54.1 (10.8)	55.0 (8.7)	0.7^a^
Disease duration in years, mean (SD)^d^	NA	3.9 (2.9)	4.2 (2.1)	0.6^a^
Time to death in years, mean (SD)^e^	NA	5.6 (2.8)	6.4 (3.3)	0.3^a^
Scanner A/B/C/D^f^	14/27/8/1	7/37/15/3	1/26/9/4	0.02^b^

Key: AD, Alzheimer's disease; FTLD, frontotemporal lobar degeneration; MMSE, Mini-Mental State Examination; NA, not applicable.

a Analysis of variance.

b Fisher exact test.

c Available in: 15 controls, 53 AD, and 28 FTLD.

d Time between first symptoms (onset) and scan.

e Time between scan and death, available in: 53 AD and 35 FTLD.

f A, B, C, D: different 1.5 T Signa GE scanners.

**Table 2:** Inter- and intrarater kappa scores (95% CI) for MTA and PA scale

Scale	Side	Interrater (*n* = 152)	Intrarater (*n* = 38)	
			Rater 1	Rater 2
MTA	LH	0.88 (0.84, 0.92)	0.90 (0.84, 0.96)	0.91 (0.84, 0.96)
	RH	0.86 (0.80, 0.90)	0.87 (0.77, 0.94)	0.83 (0.71, 0.92)
	Mean LH and RH	0.91 (0.87, 0.93)	0.90 (0.83, 0.95)	0.90 (0.83, 0.95)
PA	LH	0.83 (0.76, 0.88)	0.88 (0.77, 0.96)	0.88 (0.74, 0.96)
	RH	0.82 (0.75, 0.87)	0.88 (0.78, 0.95)	0.87 (0.74, 0.95)
	Mean LH and RH	0.84 (0.77, 0.89)	0.89 (0.79, 0.95)	0.88 (0.76, 0.96)

Key: CI, confidence interval; LH, left hemisphere; MTA, medial temporal lobe atrophy; PA, posterior atrophy; RH, right hemisphere.

**Table 3:** Volumes of hippocampus per MTA grade, and posterior cingulate gyrus per PA grade

Grade^a^	Hpc volumes per MTA grade				PCG volumes per PA grade			
	LH		RH		LH		RH	
	*n*	Mean (SD)	*n*	Mean (SD)	*n*	Mean (SD)	*n*	Mean (SD)
0 and 0.5	19	2598 (534)	18	2732 (544)	18	2449 (685)	17	2809 (459)
1 and 1.5	9	2182 (403)	12	2419 (290)	14	2197 (818)	13	2591 (798)
2 and 2.5	5	1892 (196)	7	2115 (707)	6	2021 (520)	8	1900 (540)
≥ 3	5	1985 (526)	1	1488 (0)	0	—	0	—

Mean volumes and SDs are shown in mm^3^. Key: Hpc, hippocampus; LH, left hemisphere; MTA, medial temporal lobe atrophy; PA, posterior atrophy; PCG, posterior cingulate gyrus; RH, right hemisphere.

a Mean of grades from 2 raters.

**Table 4:** Mean and SD of MTA and PA scores in control, AD, and FTLD and *p* values from Wilcoxon rank-sum test comparing distribution of scores between groups

Scale	Side	Control (*n* = 50)	AD (*n* = 44)	FTLD (*n* = 27)	Control vs. AD	Control vs. FTLD	AD vs. FTLD
		Mean (SD)	Mean (SD)	Mean (SD)	*p*	*p*	*p*
MTA	LH	0.44 (0.58)	1.43 (1.08)	2.37 (1.13)	<0.0001	<0.0001	0.002
	RH	0.37 (0.56)	1.24 (0.98)	1.37 (1.00)	<0.0001	<0.0001	0.7
	Mean LH and RH	0.41 (0.53)	1.34 (0.95)	1.87 (0.89)	<0.0001	<0.0001	0.03
PA	LH	0.77 (0.64)	1.40 (0.83)	1.13 (0.67)	0.0002	0.03	0.2
	RH	0.77 (0.68)	1.45 (0.81)	0.91 (0.65)	0.0001	0.3	0.004
	Mean LH and RH	0.77 (0.62)	1.43 (0.77)	1.02 (0.62)	0.0001	0.1	0.02

Key: AD, Alzheimer's disease; FTLD, frontotemporal lobar degeneration; LH, left hemisphere; MTA, medial temporal lobe atrophy; PA, posterior atrophy; RH, right hemisphere.

**Table 5:** Different atrophy patterns in control, AD, and FTLD

	Control	AD	FTLD
No atrophy	72%	18%	4%
MTA only	6%	18%	63%
PA only	18%	30%	7%
MTA and PA	4%	34%	26%

Shown are proportion of subjects in percent who had no atrophy, MTA only, PA only, and both MTA and PA. Key: AD, Alzheimer's disease; FTLD, frontotemporal lobar degeneration; MTA, medial temporal lobe atrophy; PA, posterior atrophy.

**Table 6:** AUCs for classification of control, AD, and FTLD

Group	Side	MTA	PA	MTA and PA	Adding PA to MTA	*p*	Adding MTA to PA	*p*
		AUC	AUC	AUC	Difference (95% CI)		Difference (95% CI)	
C vs. AD	LH	0.77	0.72	0.86	0.08 (0.01–0.16)	0.02	0.14 (0.05–0.23)	0.002
	RH	0.76	0.74	0.83	0.06 (0.00–0.16)	0.07	0.09 (0.02–0.16)	0.01
	Mean LH and RH	0.80	0.74	0.87	0.07 (0.01, 0.14)	0.03	0.13 (0.04, 0.22)	0.004
C vs. FTLD	LH	0.92	0.65	0.92	0.00 (−0.02 to 0.02)	1.0	0.28 (0.15–0.40)	<0.001
	RH	0.81	0.57	0.80	−0.01 (−0.04 to 0.03)	0.7	0.23 (0.08–0.39)	0.003
	Mean LH and RH	0.93	0.61	0.92	−0.01 (−0.03 to 0.01)	0.4	0.30 (0.17–0.44)	<0.001
AD vs. FTLD	LH	0.72	0.60	0.74	0.02 (−0.03–0.08)	0.5	0.15 (0.02–0.28)	0.03
	RH	0.53	0.70	0.72	0.19 (0.02–0.35)	0.02	0.02 (−0.05–0.08)	0.6
	Mean LH and RH	0.66	0.66	0.73	0.08 (−0.02–0.17)	0.1	0.07 (−0.03–0.18)	0.2

Data are for single and combined rating scales, as well as differences in AUCs between combined and single rating scales, 95% confidence intervals (CI) and *p* values. Key: AD, Alzheimer's disease; AUC, area under the receiver operator curve; C, control; FTLD, frontotemporal lobar degeneration; LH, left hemisphere; MTA, medial temporal lobe atrophy; PA, posterior atrophy; RH, right hemisphere.

**Table 7:** Means and SDs of MTA and PA scores in younger and older controls, EOAD and LOAD, *p* values from Wilcoxon rank-sum test comparing distribution of scores between groups

Group and comparison	MTA			PA		
	LH	RH	Mean LH and RH	LH	RH	Mean LH and RH
Younger controls (*n* = 33), mean (SD)	0.38 (0.53)	0.29 (0.43)	0.33 (0.44)	0.65 (0.58)	0.71 (0.56)	0.68 (0.53)
EOAD (*n* = 33), mean (SD)	1.29 (1.04)	1.05 (0.95)	1.17 (0.92)	1.42 (0.79)	1.45 (0.81)	1.44 (0.76)
Younger controls vs. EOAD, *p*	0.0002	0.001	0.0001	0.0001	0.0001	0.0001
Older controls (*n* = 14), mean (SD)	0.68 (0.67)	0.64 (0.77)	0.66 (0.69)	1.14 (0.66)	1.07 (0.85)	1.11 (0.72)
LOAD (*n* = 11), mean (SD)	1.86 (1.12)	1.82 (0.87)	1.84 (0.88)	1.32 (0.96)	1.45 (0.85)	1.39 (0.84)
Older controls vs. LOAD, *p*	0.01	0.003	0.002	0.7	0.3	0.5
EOAD vs. LOAD, *p*	0.1	0.02	0.04	0.7	0.8	0.7

Key: EOAD, early-onset Alzheimer's disease; LH, left hemisphere; LOAD, late-onset Alzheimer's disease; MTA, medial temporal lobe atrophy; PA, posterior atrophy; RH, right hemisphere.

**Table 8:** Different atrophy patterns in younger and older controls, EOAD, and LOAD

	Younger controls	Older controls	EOAD	LOAD
No atrophy	81%	43%	24%	0%
MTA only	3%	14%	9%	46%
PA only	12%	36%	33%	18%
MTA and PA	3%	7%	33%	36%

Data are proportion of subjects in percent who had no atrophy, MTA only, PA only, and both MTA and PA. Key: EOAD, early-onset Alzheimer's disease; LOAD, late-onset Alzheimer's disease; MTA, medial temporal lobe atrophy; PA, posterior atrophy.

**Table 9:** AUCs for classification of younger and older controls, EOAD and LOAD,

Group	Side	MTA	PA	MTA and PA	Adding PA to MTA		Adding MTA to PA	
		AUC	AUC	AUC	Difference (95% CI)	*p*	Difference (95% CI)	*p*
Younger controls vs. EOAD	LH	0.75	0.77	0.86	0.11 (0.02–0.21)	0.02	0.09 (0.01–0.17)	0.03
	RH	0.74	0.77	0.84	0.10 (0.00–0.20)	0.05	0.07 (−0.01–0.14)	0.07
	Mean LH and RH	0.77	0.78	0.89	0.11 (0.02–0.21)	0.02	0.10 (0.01–0.20)	0.03
Older controls vs. LOAD	LH	0.81	0.55	0.89	0.07 (−0.06–0.21)	0.3	0.34 (0.09–0.60)	0.01
	RH	0.85	0.61	0.87	0.02 (−0.10–0.14)	0.8	0.26 (0.04–0.48)	0.02
	Mean LH and RH	0.87	0.58	0.91	0.04 (−0.07–0.14)	0.5	0.33 (0.09–0.57)	0.01
EOAD vs. LOAD	LH	0.65	0.54	0.65	−0.01 (−0.12–0.10)	0.9	0.11 (−0.10–0.32)	0.3
	RH	0.73	0.52	0.76	0.03 (−0.07–0.13)	0.5	0.24 (0.02–0.46)	0.03
	Mean LH and RH	0.71	0.54	0.69	−0.02 (−0.10–0.07)	0.7	0.15 (−0.06–0.36)	0.2

Shown are single and combined rating scales, and differences in AUCs between combined and single rating scales, 95% confidence intervals (CI), and *p* values. Key: AUC, area under the receiver operator curve; EOAD, early-onset Alzheimer's disease; LOAD, late-onset Alzheimer's disease; MTA, medial temporal lobe atrophy; PA, posterior atrophy.
