# Supplementary material for: Posterior cerebral atrophy in the absence of medial temporal lobe atrophy in pathologically-confirmed Alzheimer's disease
Source: Neurobiol Aging. 2012 Mar;33(3):627.e1–627.e12. doi: 10.1016/j.neurobiolaging.2011.04.003 (PMC3657170; doi:10.1016/j.neurobiolaging.2011.04.003)
Supplement: Supplementary Table 1 [file mmc1.doc]

**SUPPLEMENTARY MATERIAL**

**Table 1:** Demographics of AD patients according to atrophy pattern.

|  | **No atrophy** | **MTA only** | **PA only** | **MTA & PA** | **p value** |
| --- | --- | --- | --- | --- | --- |
| **N** | 8 | 8 | 13 | 15 | - |
| **Age, mean (SD) in years** | 63.3 (5.5) | 66.7 (11.6) | 60.6 (7.0) | 61.0 (10.0) | 0.3 |
| **Gender % male** | 63% | 75% | 46% | 67% | 0.6 |
| **MMSE, mean (SD) ^†^** | 16.8 (6.7) | 18.8 (6.3) | 17.0 (6.1) | 15.9 (7.9) | 0.8 |
| **Age of onset** | 58.4 (3.6) | 63.4 (12.2) | 56.9 (6.9) | 57.6 (10.2) | 0.3 |
